# Executive Control and Striatal Resting-State Network Interact with Risk Factors to Influence Treatment Outcomes in Alcohol-Use Disorder

**DOI:** 10.3389/fpsyt.2017.00182

**Published:** 2017-09-25

**Authors:** Milky Kohno, Laura E. Dennis, Holly McCready, William F. Hoffman

**Affiliations:** ^1^Department of Psychiatry, Oregon Health & Science University, Portland, OR, United States; ^2^Department of Behavioral Neuroscience, Oregon Health & Science University, Portland, OR, United States; ^3^Methamphetamine Abuse Research Center, Portland, OR, United States; ^4^Mental Health and Clinical Neurosciences Division, Portland, OR, United States; ^5^Research Service Veterans Affairs Portland Healthcare System, Portland, OR, United States

**Keywords:** alcohol-use disorder, relapse, treatment, craving, mesocorticolimbic, resting-state fMRI

## Abstract

Alterations within mesocorticolimbic terminal regions commonly occur with alcohol use disorder (AUD). As pathological drug-seeking behavior may arise as a consequence of alcohol-induced neuroadaptations, it is critical to understand how such changes increase the likelihood of relapse. This report examined resting-state functional connectivity (RSFC) using both a seed-based and model-free approach in individuals in treatment for AUD and how dysregulation of network connectivity contributes to treatment outcomes. In order to provide a mechanism by which neural networks promote relapse, interactive effects of mesocorticolimbic connectivity and AUD risk factors in treatment completers and non-completers were examined. AUD group showed stronger RSFC between striatum, insula, and anterior cingulate cortex than controls. Within the AUD group, non-completers compared to completers showed enhanced RSFC between (1) striatum–insula, (2) executive control network (ECN)–amygdala, and (3) basal ganglia/salience network and striatum, precuneus, and insula. Completers showed enhanced RSFC between striatum-right dorsolateral prefrontal cortex. Furthermore, completers and non-completers differed in relationships between RSFC and relapse risk factors, where non-completers exhibited positive associations between craving intensity and RSFC of striatum–insula and ECN–amygdala. These findings provide evidence for interactions between corticolimbic connectivity in AUD and craving and establish an important link between network connectivity and dynamic risk factors that contribute to relapse. Results demonstrate that relapse vulnerability is attributed to craving dysregulation manifested by enhanced connectivity in striato-limbic regions and diminished corticostriatal connectivity.

## Introduction

Identification of neural phenotypes related to risk for relapse is important for understanding the nature of alcohol-use disorder (AUD) and its response to treatment. AUD is associated with widespread neural adaptations, but how these changes correspond to phenotypes that promote relapse is unclear. Cognitive domains, such as executive control and reward processing, likely interact to contribute to the maintenance of alcohol seeking and problem drinking. Enhanced reward sensitivity and the motivational drive that promote reward-seeking behavior may in part result from impairments in executive control. Similarly, an increase in attentional bias to salient drug stimuli may enhance craving and subsequent relapse (1). The behavioral sequelae of alterations in the balance of cognitive control and reward-seeking behavior are key features of substance-use disorders and common barriers to treatment success.

Neuroimaging studies have shown functional neuroadaptations in key regions and networks responsible for cognitive control and reward salience during decision-making and cue-induced craving (2, 3). These functional deficits are, however, task specific, and it is, therefore, important to understand if this neural dysregulation is a manifestation of adaptations in intrinsic connectivity associated with AUD. Studies of resting-state functional connectivity (RSFC) have contributed new insights into drug-related adaptations through identification of abnormalities in the functional organization of brain systems. In AUD, however, the results are mixed. Some studies have reported higher connectivity within the cognitive control network and lower connectivity in the reward network compared to controls (4, 5), while others suggest that alcohol-dependent individuals exhibit greater connectivity in networks comprised of striatum, amygdala, and insula (6). Network efficiency has also been examined and alcohol-dependent individuals show weaker within reward-motivational network connectivity and expanded executive control network (ECN) connectivity compared to controls.

As RSFC impairments in AUD are still unclear, in part, due to differences in participant demographics across studies along with different approaches in identifying RSFC networks (7), this study used two methods to investigate RSFC. For a specific targeted examination of dopaminergic terminal regions, a seed-based analysis was used. This study examined whole-striatal connectivity, as alcohol-motivated behaviors are likely to depend on both ventral and dorsal striatum, where the ventral striatum is involved in stimulus–reward associations and the dorsal striatum is critical in goal-directed and compulsive alcohol-seeking behavior (8). In addition, animal models show that high alcohol drinking rats have lower markers of dopamine signaling in dorsal and ventral striatum (9) and that cue-induced dopamine release was seen in both ventral and dorsal striatum (10) demonstrating a role for whole striatal dopamine signaling in action selection and control over self-administration. This study also implemented an independent component analysis (ICA) as a model-free approach. As the ECN is implicated in cognitive control and goal-directed attention and the salience/reward network is proposed to bias network attention toward rewarding and motivating stimuli, these networks are thought to underlie psychological dysfunctions associated with addiction in reward as well as affective and cognitive processing (11). This study, therefore, examined executive control and reward/salience networks for large-scale network dynamics in AUD.

As RSFC may provide a marker for deficits in brain function, studies have examined how abnormalities of RSFC relate to neuropsychological assessments of cognition, anxiety, and depression or how it is affected by alcohol use. For example, a measure of impulsivity is associated with greater RSFC between the salience network and the ECN in AUD (6). Greater and more expanded ACC–frontostriatal connectivity and expanded network connectivity is positively associated with visuospatial working memory and slower perceptual motor processing, respectively, while measures of depression and anxiety are related to restricted and expanded connectivity of reward and ECNs (12). While these findings provide a theoretical framework for how neural network adaptations in AUD and the relationship to cognitive or neuropsychiatric constructs may lead to relapse, the interactive effects of RSFC, risk factors for relapse, and treatment outcomes have not been explored. For example, a study reported that relapse was associated with reduced baseline RSFC between a nucleus accumbens seed and insula and dorsolateral prefrontal cortex (DLPFC), which was negatively related to alcohol-use measures and performance on an affective Go/No-Go task; the link, however, between RSFC abnormalities and specific addiction-related phenotypes that contribute to relapse was not directly examined (5).

Craving is a critical component in AUD and has been shown to predict relapse in individuals undergoing residential addiction treatment (13–15). Moreover, individuals with higher craving intensity during treatment endorsed more alcohol-related problems and not only had an increase in the likelihood of relapse but relapsed more quickly (15). In addition, these relapse predictors interact, such that the likelihood of alcohol consumption is associated with higher craving and greater loss of control (16). These studies suggest that craving and loss of control lie along a continuum of alcohol severity and vulnerability to relapse. Task-based fMRI studies have addressed factors important in the maintenance of addiction and have examined neural responses during cognitive control and craving reactivity. Investigating whether abnormal intrinsic activity occurs in the absence of tasks, however, may provide an important brain indicator for the propensity of craving dysregulation and impaired control over alcohol use. The examination of neural networks in the context of these meaningful outcome measures and the mechanisms contributing to treatment-related domains would provide a better account of the inter-relationships of brain function, psychological states, and outcome measures.

This study, therefore, examined how RSFC differs between individuals who successfully can and cannot complete treatment for AUD and how differences are attributed to AUD characteristics that are predictive of relapse. Striatal-seed based and independent component analyses were used to identify differences in network structure between treatment completing and non-treatment completing individuals with AUD. We expected that non-completers would have weaker RSFC in regions and networks responsible for cognitive control and stronger RSFC in reward/salience networks. We also hypothesized that craving would be negatively associated with RSFC of cognitive control networks but positively with RSFC of reward/salience networks.

## Materials and Methods

### Participants

Forty-three volunteers diagnosed with AUD, recruited from the VA Portland Healthcare System (VAPORHCS) and community substance abuse treatment programs and 26 healthy controls recruited with online advertisements, completed the study and provided written informed consent, as approved by the VAPORHCS and Oregon Health & Science University Institutional Review Boards. Exclusion criteria, determined by medical history and laboratory blood tests were: systemic, neurological, cardiovascular, or pulmonary disease, head trauma with loss of consciousness, MRI contraindications, use of antidepressants or medications known to have dopaminergic mechanisms (e.g., antipsychotics, antidepressants, antiparkinsonian agents), sedative-hypnotics (barbiturates, benzodiazepines, zolpidem), or anticholinergics. Past or Current Axis I diagnoses, other than depression or PTSD, nicotine dependence for either group and alcohol dependence for the Alcohol group, assessed with the Structured Clinical Inventory for DSM-IV-TR, were exclusionary. Ten participants (9 AUD and 1 control) were excluded based on these criteria, and data from 2 AUD and 2 control participants were excluded for excessive motion. Final analyses included data from 43 AUD and 26 control participants. Participants received $50 gift cards to a local retail chain for completing visit 1 (screening) and visit 2 (scan) and the AUD subjects received $20 for each follow-up visit.

The Alcohol group were primarily recruited from residential treatment facilities and reported abstention from alcohol for 1–4 weeks before scanning. Following the baseline scan, AUD subjects maintained a weekly drinking diary for 3 months and were asked to report any alcohol or drug use, which was verified by their treatment provider. The diary, medical records, and information from the subject’s treatment provider were assessed at monthly follow-up visits. Participants (*n* = 16), who completed the 3-month study without relapse or with minor lapses, as defined by a maximum of two consecutive days of non-heavy drinking (<5 drinks per day) were considered Completers. Non-completers (*n* = 27) included subjects who dropped out of the study or reported relapse, defined as at least one or more days of heavy drinking (>5 drinks per day) (17).

### Measures of Addiction and Craving Severity

Craving was assessed at the day of the scan with the Visual Analog Scale measuring craving intensity on a scale of 0–100.

### MRI Imaging Acquisition

Imaging was performed on a 3 Tesla Siemens TIM Trio MRI scanner. A localizer scan was acquired to guide slice alignment during anatomical and functional scans. A T_2_*-weighted echo-planar image (EPI) was acquired (24 slices, 4 mm thick, gap width = 1 mm, TR/TE/α = 2,000 ms/40 ms/80°, matrix = 128 × 128, FOV = 240 mm^2^, 170 volumes, in-plane pixel size of 1.875 mm^2^), while subjects stared at a white cross on a black screen. One high-resolution T1-weighted anatomical magnetically prepared rapid acquisition gradient echo (MPRAGE; 144 slices 1 mm thick, TR/TE/TI/α = 2,300 ms/4.38 ms/1,200 ms/12°, FOV = 208 mm × 256 mm) was acquired for co-registration with functional images and statistical overlay.

### Resting-State fMRI Image Processing: Seed-Based Approach

Image analysis was performed using FSL 5.0.2.1 (www.fmrib.ox.ac.uk/fsl). Images were realigned to compensate for motion (18), and high-pass temporal filtering (100 s) was applied. Data were skull-stripped and spatially smoothed (5-mm FWHM Gaussian kernel). Images were further pre-processed to include additional nuisance regressors: average signal of cerebrospinal fluid and white-matter, and two metrics of motion-related artifact—motion scrubbing with frame-wise displacement and a combination of the temporal derivative of the time series and root-mean-squared variance over all voxels (19). Global signal regression was not applied. The EPI images were registered to the high-resolution MPRAGE image and then into standard Montreal Neurological Institute space, using a 12-parameter affine transformation. An anatomically defined region of interest (ROI) from the Harvard-Oxford Subcortical atlas of the whole striatum was used as the seed. The seed was transformed into each subject’s native space by applying the inverted transformation matrix of EPI to MPRAGE to standard space. The mean time series across all voxels within the striatum seed from preprocessed images were used as covariates in separate whole-brain, voxel-wise resting-state correlation analyses.

### Analysis

Whole-brain voxel-wise analyses of striatum RSFC was conducted. Completers and non-completers were combined and compared to controls to investigate the RSFC differences in alcohol-use disorder. Separate analyses within the AUD group were conducted to compare completers to non-completers and to examine the linear relationship with alcohol-use status. Non-completers, completers, and healthy controls were modeled separately. For within- and between-group mixed-effects analyses, all whole-brain fMRI statistics were corrected for multiple comparisons by using cluster-correction with voxel height threshold of *Z* > 2.3 and cluster significance of *P* < 0.05. As there were significant differences in age, sex, and years of education between the Alcohol and control groups, these variables were modeled as nuisance covariates. Smoking status, however, was not used as a nuisance covariate. As there were very few healthy controls who smoked, the distribution of the effects of smoking status would, therefore, not be sufficient to accurately model and control for smoking status.

### Model-Free Resting-State fMRI Image Processing: ICA

Resting-state data from completers and non-completers were submitted to an ICA using Multivariate Exploratory Linear Optimized Decomposition into Independent Components with FSL. Data were preprocessed and registered as described above. The number of components generated was not restricted, and 86 independent components were identified with a free estimation for the number of components. In order to investigate the similarities in ICA outputs, the components of interest were selected by cross correlating the spatial maps of our independent components with that of the resting-state template (20). As the executive control and basal ganglia/salience networks are highly relevant in studies of addiction (7, 21, 22), components that most highly correlated (*r* = 0.3, 0.6) with these networks were chosen for further group analyses.

### Analysis

Dual regression (23) was used to decompose the networks into spatial maps to be used as regressors in a general linear model to find the average time course of the BOLD signal across each subject for each network. The time courses were then variance-normalized and used as a set of temporal regressors to test differences between completers and non-completers in the executive control and basal ganglia/salience networks with 10,000 non-parametric permutations. A family-wise error correction for multiple comparisons was performed, implementing threshold-free cluster enhancement using a significance threshold of *p* < 0.025 to correct for the number of components tested.

### Analysis of the Relationship between RSFC and Measures of Addiction Severity and Craving

Connectivity values (regression coefficients) were extracted from functional ROIs that showed significant differences between groups. These values correspond to the strength of functional connectivity of each ROI with the striatum. A general linear model was used to examine the relationship between connectivity values and VAS craving scores. Connectivity values were entered as an independent variable in ANCOVA with outcome measure being craving scores, separately. The models tested the main effects and the interaction between group and RSFC.

## Results

The healthy control group included 26 subjects (12 women/14 men, 2 smokers, 34.19 ± 11.37 years old). They reported no heavy or daily use of alcohol or any other drug use. The Alcohol group included 43 alcohol-dependent subjects (10 women/33 men, 27 smokers, 41.93 ± 9.57 years old) and had abstained from alcohol use for 25.71 ± 12.46 days before scanning. They had used alcohol for 20.69 ± 10.39 years and reported 16.43 ± 8.23 standard drinks per day. There were no significant differences between Completers and Non-completers in age, sex, years of education, alcohol-use variables, or in the frequency of cigarette use but significant differences between controls and the AUD group (completers and non-completers combined) were seen in age, sex, years of education, and smoking status (*p* < 0.05) (Table 1).

**Table 1:** Characteristics of research participants.

	Completers (*n* = 16)^a^	Non-completers (*n* = 27)^b^	Healthy controls (*n* = 26)
Age (years)^c^	41.38 ± 8.61	42.26 ± 10.24	34.19 ± 11.37^d^
Sex (no. of male)	14	19	14^d^
Education (years)	12.94 ± 1.53	12.63 ± 1.97	13.69 ± 2.07^d^
Alcohol use			
Years of use	20.19 ± 10.22	20.98 ± 10.67	
Standard drinks per day	18.22 ± 9.87	15.43 ± 7.18	
Days abstinent prior to MRI	29.38 ± 10.67	23.46 ± 13.14	
Tobacco use (no. of smokers)	10	17	2^d^
Cigarettes per day	11.30 ± 5.64	11.86 ± 7.42	23.33 ± 15.27^d^

*^a^n = 3 lapsed*.

*^b^n = 16 lost to follow-up*.

*^c^Data shown are means ± SD*.

*^d^Significant differences between controls and alcohol-use disorder groups combined (*p* < 0.05)*.

*No significant differences between alcohol-use disorder groups in demographic or drug use variables*.

### Resting-State fMRI Image Processing: Seed-Based Approach

**Figure 1:**
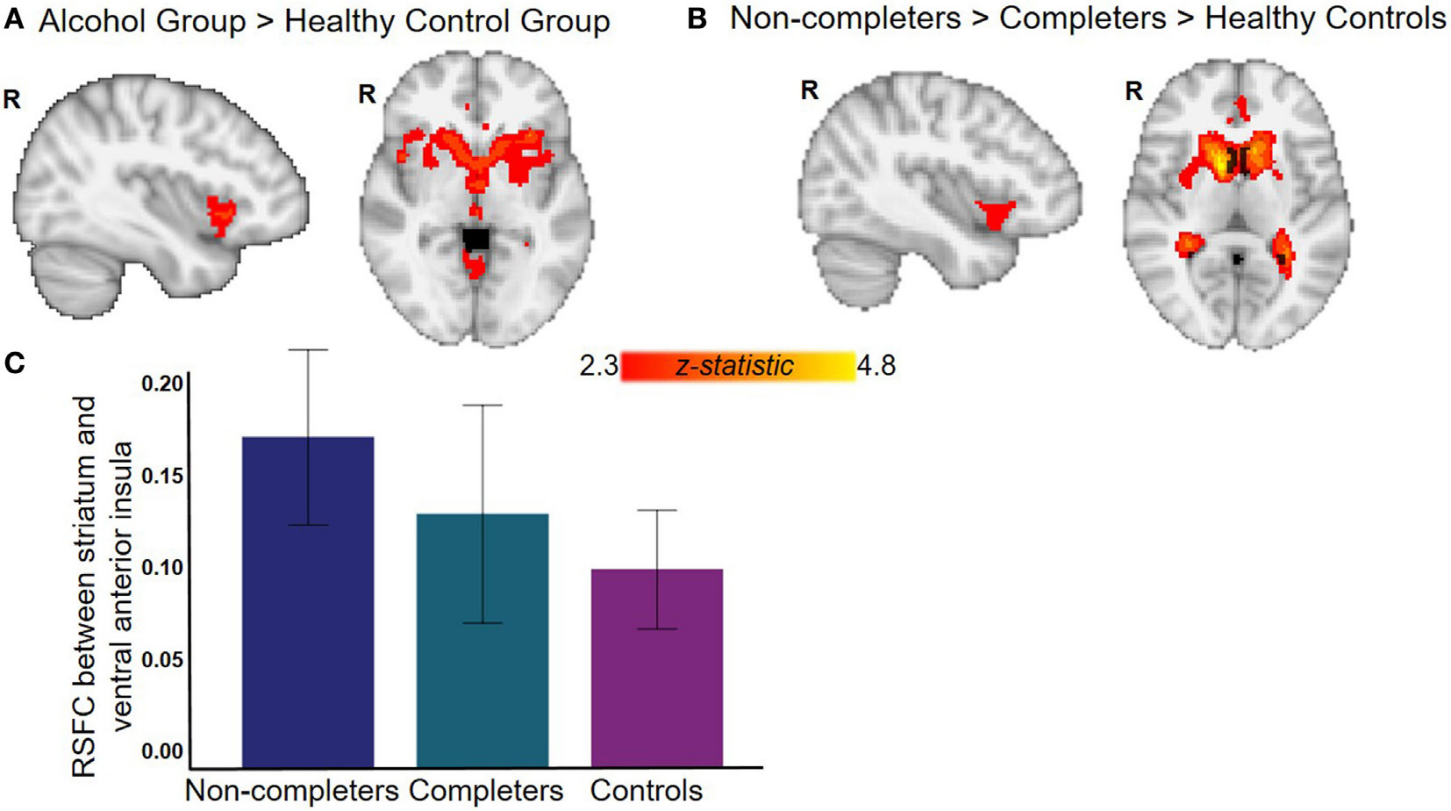
Resting-state functional connectivity (RSFC) with striatum seed. **(A)** Alcohol group (completers and non-completers combined) exhibit greater striatal RSFC within striatum and with right bilateral ventral anterior insula and anterior cingulate (see Table 2 for complete list of regions). **(B)** Relationship with alcohol-use status whereby non-completers have greater striatal RSFC than completers who have greater striatal RSFC than controls. **(C)** Graph displays RSFC between striatum and ventral anterior insula for each group (for illustrative purposes, whole-brain results corrected at *p* < 0.05). All analyses with controls were corrected for age, sex, years of education, and whole-brain multiple comparisons (*p* < 0.05).

**Figure 2:**
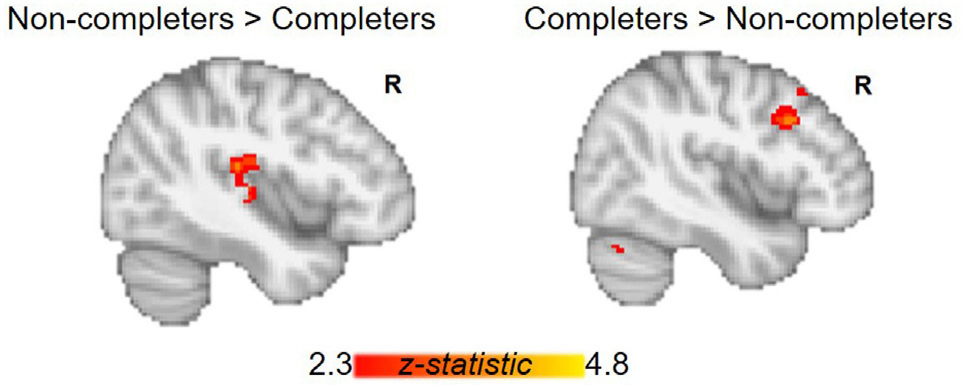
Group differences between alcohol-use groups in resting-state functional connectivity (RSFC) of the striatum. Within the alcohol-use group, non-completers exhibit greater RSFC between striatum and posterior insula, while completers show greater RSFC between striatum and right middle frontal gyrus. Corrected for whole-brain multiple comparisons (*p* < 0.05).

The AUD group (completers and non-completers combined) compared to controls exhibited greater connectivity within the striatum and between striatum and insula, inferior and superior frontal gyri, anterior and paracingulate cortices, and cerebellum but weaker connectivity between striatum and inferior temporal gyrus and occipital cortex (*p* < 0.05, whole-brain corrected) (Figure 1; Table 2). Within the AUD group, non-completers exhibited greater connectivity between striatum and posterior insula, superior temporal gyrus, brain stem, cuneus and thalamus, and weaker connectivity between striatum and middle frontal gyrus and cerebellum (*p* < 0.05, whole-brain corrected) (Figure 2; Table 2). The analyses including completers, non-completers, and controls showed a relationship with alcohol-use severity, such that, connectivity was greater in non-completers than completers and in completers than controls between striatum and ventral anterior insula (*p* < 0.05, whole-brain corrected) (Figure 1).

**Table 2:** Brain regions that exhibited differences in striatal RSFC between groups and within alcohol-use disorder groups.

Brain region	Cluster size (voxels)	*x*^a^	*y*	*z*	*z*-statistic
**Alcohol group > healthy controls**					

Cluster #1^b^	9,727				
Caudate (L/R)		12	6	16	8.34
Putamen (L/R)		28	6	6	4.23
Ventral anterior insula (L/R)		−36	18	−4	4.17
Inferior frontal gyrus (L/R)		60	22	16	3.91
Cluster #2	4,532				
Paracingulate cortex		−2	40	34	5.46
Superior frontal gyrus		2	50	40	5.36
Anterior cingulate cortex		−2	40	10	4.09
Cluster #3	946				
Cerebellum		12	−48	−26	3.82

**Healthy controls > alcohol group**					

Cluster #1^b^	16,902				
Inferior temporal gyrus (L/R)^c^		54	−58	−2	6.66
Lateral occipital cortex (L/R)		−44	−64	−4	6.04

**Completers > non-completers**					

Cluster #1^b^	823				
Cerebellum (L/R)^c^		12	−78	−26	3.84
Cluster #2	320				
Middle frontal gyrus (R)		46	18	34	3.92

**Non-completers > completers**					

Cluster #1^b^	736				
Superior temporal gyrus (L)		−56	−8	2	4.40
Insular cortex (L)		−36	−22	16	3.97
Parietal operculum (L)		−48	−14	18	3.47
Postcental gyrus (L)		−62	−18	24	3.31
Cluster #2	571				
Cuneal cortex (L/R)		16	−78	28	4.89
Temporal fusiform cortex (R)		28	−50	−8	3.51
Thalamus		−8	−26	4	3.48
Brainstem		4	−36	−12	3.28
Cluster #3	321				
Occipital cortex		50	−76	0	4.39

*Z-statistic maps were thresholded using cluster-corrected statistics with a height-threshold of Z > 2.3 and cluster-forming threshold of p < 0.05*.

*^a^x, y, z reflect coordinates for peak voxel or for other local maxima in MNI space*.

*^b^Clusters are numbered and presented in order of decreasing size*.

*^c^L or R refers to left or right hemisphere*.

### Model-Free Resting-State fMRI Image Processing: ICA

**Figure 3:**
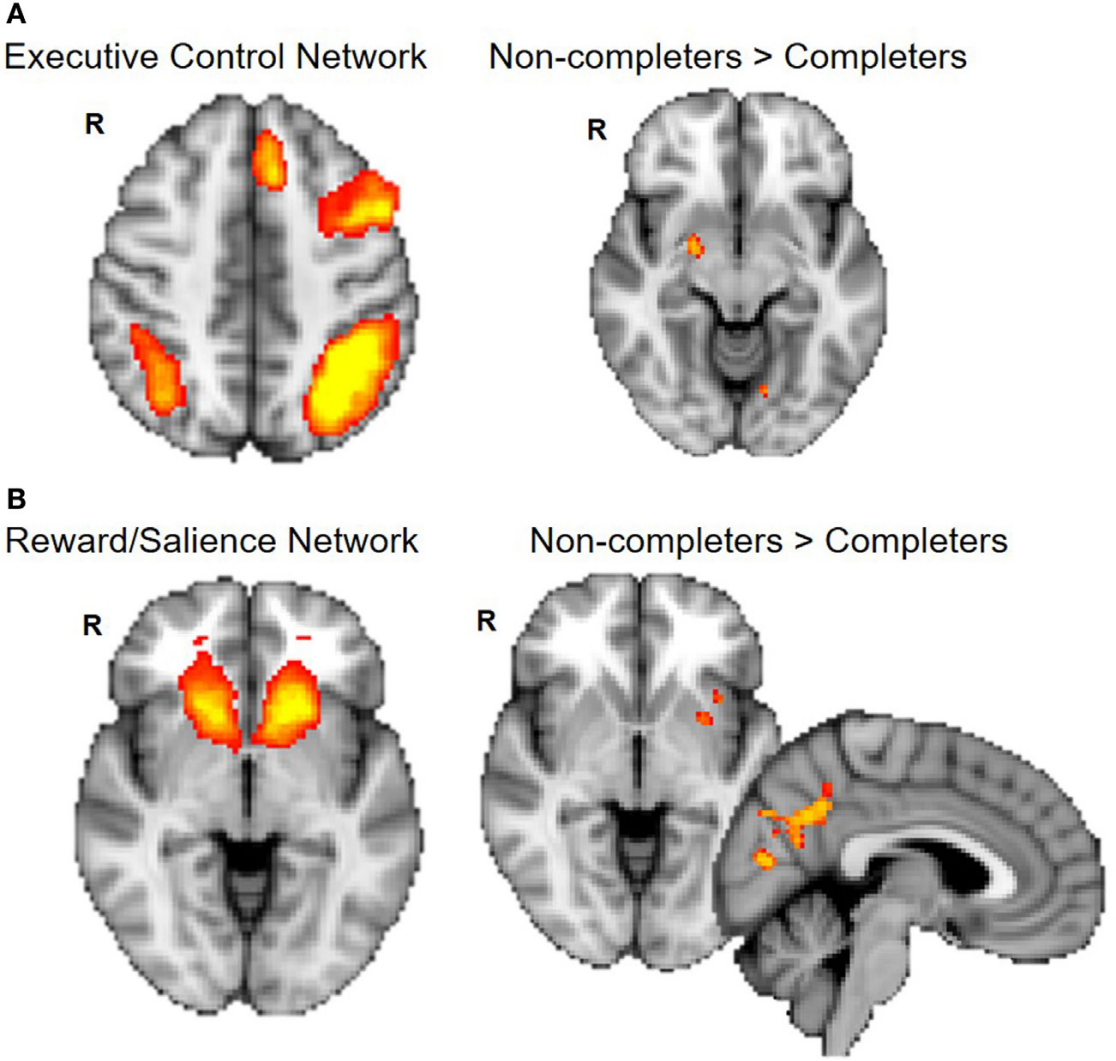
Group differences in connectivity within executive control and reward/salience networks. Non-completers compared to completers exhibit greater connectivity of the executive control network and amygdala **(A)** and between reward/salience network and putamen, insula, and precuneus cortex **(B)**. Dual regression analyses with 10,000 permutations were corrected for two networks tested (*p* < 0.025).

Non-completers exhibited greater connectivity between the ECN (frontal and parietal cortices) and right amygdala compared to completers [*p* < 0.025, Threshold Free Cluster Estimation (TFCE)]. Non-completers also showed greater connectivity between the reward/salience network (striatum, ACC, and insula) and insula, putamen, and precuneus (*p* < 0.025, TFCE) (Figure 3).

### Analysis of the Relationship between RSFC and Craving

**Figure 4:**
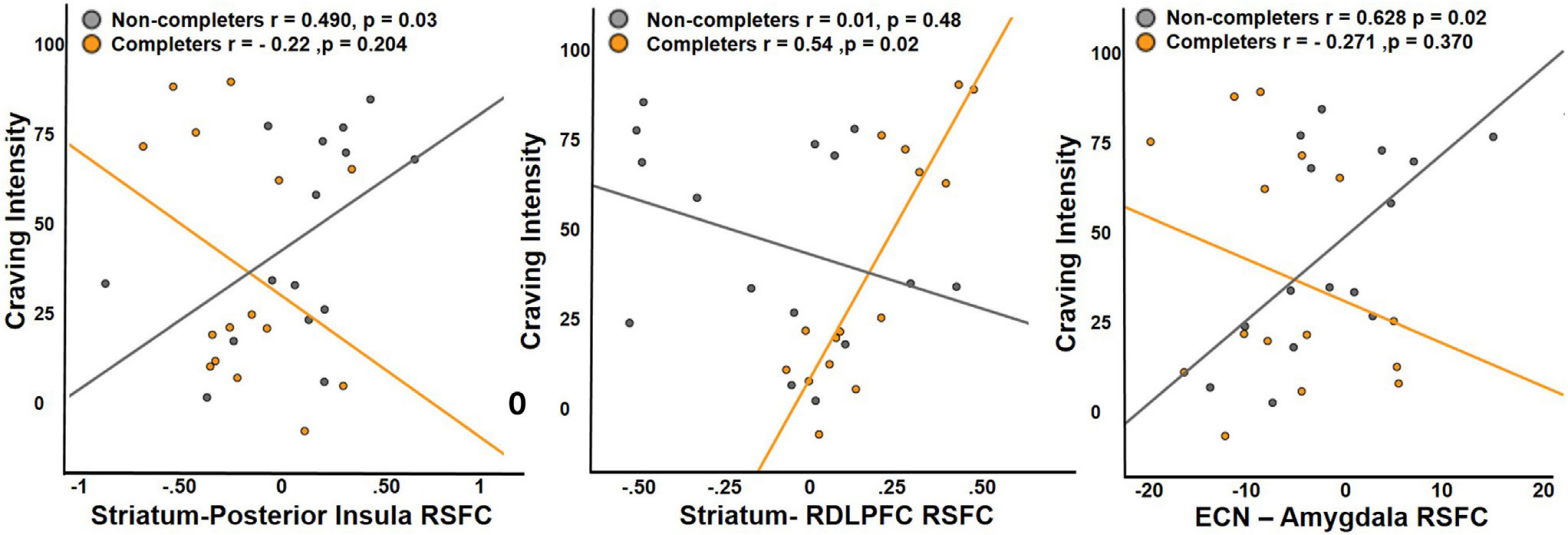
Relationship between craving and RSFC. Non-completers exhibit a significant positive relationship with craving intensity and RSFC of striatum and posterior insula, while completers exhibit a significant positive relationship with RSFC of striatum and dorsolateral prefrontal cortex. Executive control network and amygdala connectivity was positively associated with craving intensity in the non-completers and the relationship was slightly negative in treatment completers. Posterior insula and RDLPFC were functionally defined region of interest (ROIs) from the analysis comparing the two groups, while right amygdala was an anatomically defined ROI.

Completers and non-completers did not significantly differ in measures of craving. The effects of connectivity between striatum and regions that display significant group differences (posterior insula, ventral anterior insula, and right middle frontal gyrus) on craving were examined. Examination of the main effects of group and connectivity and their interaction revealed a significant main effect of striatum–right DLPFC connectivity on craving intensity and a significant interaction by group (interaction: β = 177.32, *t* = 4.642, *p* < 0.001) (Figure 4). Completers exhibited a positive relationship between striatum–right DLPFC connectivity and craving intensity (*r* = 0.54), while non-completers showed a negative relationship (*r* = −0.37). An interaction of group on craving and posterior insula connectivity was observed (interaction: β = 73.68, *t* = 2.02, *p* = 0.05), where the non-completers exhibited a positive relationship (*r* = 0.49) and completers exhibited a negative relationship (*r* = −0.22) (Figure 4).

For ICA network connectivity, we found group interactions in the relationship between ECN–amygdala RSFC and craving (interaction: β = 3.597, *t* = 2.213, *p* < 0.037) (Figure 4). Non-completers showed a positive relationship (*r* = 0.628) between craving intensity and ECN–amygdala RSFC, while the relationship was negative in completers (*r* = −0.271).

## Discussion

Intrinsic RSFC has been proposed as a potential biomarker for the understanding of addiction (7), and specifically in the maintenance of addictive behaviors in AUD (7). While studies using only either seed-based or model-free RSFC approaches have reported mixed results, this study used both approaches (seed-based and ICA) to identify network structure in AUD. In addition, this study focused on seed-based and network-wide connectivity as a function of craving and treatment outcomes. Our results provide insights into the interactions between cortico-striato-limbic connectivity and how connectivity of these neural networks directly translate to dynamic risk factors that contribute to relapse.

Consistent with preclinical studies providing evidence for alcohol-induced neuroadaptations in corticolimbic circuits (24–26), our results show that the AUD group, compared to controls, displayed greater connectivity within the striatum and between the striatum, insula, and anterior cingulate cortex. Although results contrast with a report of weaker RSFC between ventral and dorsal striatum in long-term abstainers compared to controls (27), our results are consistent with findings that long-term abstainers compared to controls have greater RSFC between ventral striatum and insula (28). Our findings also agree with reports that AUD patients have greater connectivity within networks comprised of striatum, amygdala, and insula (6) and with functional task-based studies showing that alcohol use is associated with greater activation in striatum, insula, and anterior cingulate cortex (3, 29, 30). Taken together, our results are consistent with reward-centric models of addiction (31, 32), where repeated drug exposure induces long-lasting synaptic plasticity and sensitization of striatal and limbic regions to drug cues (33). Neuroadaptations in this circuitry are thought to both promote drug dependence and contribute to relapse (33) perhaps through strengthened connections between insula and striatum that drive craving and motivated drug-seeking behavior (34). The attenuation of RSFC between striatum and insula as a function of problematic drinking (controls < treatment completers < treatment non-completers) support this view and suggests that striato-limbic sensitization establishes a bias toward drug-seeking behavior (35) and that neuroadaptations in this circuit may underlie the likelihood of relapse.

Our results support a model in which executive and salience network and their interconnections play a critical role in integrating cognitive and motivational processes in the maintenance of addiction (36). AUD and other substance-use disorders affect frontostriatal neural systems, leading to an imbalance between circuits important for cognitive control and those involved in reward seeking, thus producing deficits in craving regulation and relapse vulnerability (37–39). Indeed, meta-analyses of craving studies consistently show that drug cues elicit activation in striatum, amygdala, and anterior cingulate cortex/ventromedial prefrontal cortex (40). Furthermore, neural responses to drug cues, including in the striatum, insula, and amygdala correlate with subsequent drug use (40–43).

As the PFC has a modulatory effect on subcortical activity (44, 45), regulation of striato-limbic and striato-cortical reactivity may attenuate craving and promote abstinence. Activation in the DLPFC reduces craving, while activation of the ventral striatum increases craving and show a modulating effect of the DLPFC (40). As lesions to corticostriatal projections shift decisions related to reward contingencies in rodents (46) and neurocomputational models indicate that PFC activity can directly override striato-limbic representations of reinforcement value (44), the inverse relationships between completers and non-completers in connectivity and craving may reflect differences in craving regulation through functional connectivity of prefrontal and subcortical striatal and limbic networks. Thus, our findings are consistent with a model that depicts DLPFC (and the ECN) as critical in craving regulation and cognitive control *via* modulation of the activity in striato-limbic regions. We extend these findings by providing evidence for a link between craving and the intrinsic connectivity of salience and ECNs and the interactive effects on treatment outcome.

Support for an association between circuit abnormality and relapse risk comes from the direct comparisons between successful treatment completers and non-completers. Non-completers exhibit greater RSFC between striatum and insula but weaker corticostriatal connectivity, which is consistent with preclinical studies that show engagement of frontal–striatal, frontal–mesencephalic, and amygdala–mesencephalic pathways is necessary for the reinstatement or drug-seeking behavior (47, 48). In humans, these circuits have also been implicated in drug craving (49). In AUD, specifically, neural responses to alcohol cues and craving for alcohol have been associated with activation of amygdala, ventral striatum, and insula (3, 29, 30). Although alcohol craving has been reported to be negatively associated with RSFC between ventral striatum and amygdala (12) and another study showed differences in RSFC as a function of days abstinent, the link between symptomatic features of relapse and RSFC in the context of treatment was not examined. We extend these results and demonstrate that RSFC between striatum, DLPFC, and insula and between executive control regions and amygdala are associated with alcohol craving and that these relationships differ as a function of relapse vulnerability. Specifically, we find increased craving associated with increased striato-limbic RSFC in non-completers, while greater craving is associated with corticostriatal RSFC in treatment completers. These findings suggest that for successful abstinence, corticostriatal connectivity is required to downregulate striato-limbic signaling to counter intense cravings.

Differential responses to rewards and drug-cues may guide goal-directed behavior through mesocorticolimbic signaling pathways. Considering this possibility, the ability to remain in control of alcohol use would require a balance between behavioral control and alcohol-seeking behavior, and this balance may be altered by frontal cortical regulation of ventral-limbic response to craving. Our findings are in line with this notion and the group interactions of the ECN–amygdala RSFC and craving may identify a crucial difference between individuals who can and cannot maintain sobriety. As extended network coherence may represent neural network deficiency (12), RSFC of ECN expanding to amygdala and association with enhanced craving in non-completers suggest that the efficiency of neural networks responsible for executive control is diminished. Thus, the results of this study show that functional connectivity of frontal, limbic, and striatal regions mediates cognitive control over craving dysregulation and are important biomarkers for relapse risk.

### Limitations

Limitations associated with self-report measurements of craving warrant mentioning. In this study, we examined baseline craving intensity but little is known about the temporal dynamics of these symptoms (50). In addition, this study examined how baseline measures reflect treatment outcomes and more studies examining the longitudinal changes in RSFC and correspondence to craving are needed to understand the trajectory of brain function and relapse. This study included participants who did not complete treatment and examining longitudinal changes would have benefited this study and provided a better account of differences that contribute to relapse. The examination of craving and RSFC were conducted on clusters that showed significant group differences, however, after controlling for four tests, only the relationship with striatal-DLPFC RSFC survived Bonferroni multiple comparison correction. Perhaps, using a more specific alcohol craving questionnaire rather than the VAS to assess craving may reduce variability. Controls in this study were primarily non-smokers and although tobacco use did not differ within the AUD group and there was a significant linear trend between non-completers, completer, and controls, future studies would be required to dissociate the effects of smoking from alcohol use when examining AUD and controls. In addition, although differences in other demographic factors were used as nuisance covariates, it should be noted that modeling these factors may not be sufficient in controlling for the heterogeneity between AUD and control groups. Last, although this study was unable to examine sex differences or sex by group interactions, sex differences have been shown in incidence, prevalence, and outcomes for treatment (51), and it is important to examine how sex may mediate differences in relapse risk in future studies.

## Conclusion

This study provides new evidence that RSFC interacts with relapse risk factors that contribute to treatment outcomes. We extend previous results showing that cravings are associated with intrinsic connectivity of the cortico-striato-limbic network. Identifying mechanisms to attenuate craving through upregulation of ECNs and downregulation of reward/salience networks may prove useful for developing interventions to prevent relapse.

## Ethics Statement

This study was carried out in accordance with the recommendations of the Institutional Review Boards at OHSU and VAPORHCS with written informed consent from all subjects in accordance with the Declaration of Helsinki. The protocol was approved by the Institutional Review Boards at OHSU and VAPORHCS. This study was performed under a Certificate of Confidentiality from the NIAAA.

## Author Contributions

MK conceptualized this approach, performed the final analyses, and wrote the MS. LD and HK collected these data, contributed to writing the MS, and performed initial data analyses with the neuroimages. WH was the PI on the project, conceptualized the approach, supervised the collection and analysis of data, and contributed to the writing of the MS.

## Disclaimer

The contents of this paper do not represent the views of the U.S. Department of Veterans Affairs or the United States Government.

## Conflict of Interest Statement

The authors declare that the research was conducted in the absence of any commercial or financial relationships that could be construed as a potential conflict of interest.
